# Transcription Initiation Activity Sets Replication Origin Efficiency in Mammalian Cells

**DOI:** 10.1371/journal.pgen.1000446

**Published:** 2009-04-10

**Authors:** Joana Sequeira-Mendes, Ramón Díaz-Uriarte, Anwyn Apedaile, Derek Huntley, Neil Brockdorff, María Gómez

**Affiliations:** 1 Instituto de Microbiología Bioquímica, CSIC/Universidad de Salamanca, Edificio Departamental, Salamanca, Spain; 2 PhD Programme in Experimental Biology and Biomedicine, Centre for Neuroscience and Cell Biology, University of Coimbra, Coimbra, Portugal; 3 Centro Nacional de Investigaciones Oncológicas, Madrid, Spain; 4 Clinical Sciences Centre, Medical Research Council, Hammersmith Hospital, London, United Kingdom; 5 Centre for Bioinformatics, Faculty of Natural Sciences, Imperial College London, London, United Kingdom; Medical Research Council Human Genetics Unit, United Kingdom

## Abstract

Genomic mapping of DNA replication origins (ORIs) in mammals provides a powerful means for understanding the regulatory complexity of our genome. Here we combine a genome-wide approach to identify preferential sites of DNA replication initiation at 0.4% of the mouse genome with detailed molecular analysis at distinct classes of ORIs according to their location relative to the genes. Our study reveals that 85% of the replication initiation sites in mouse embryonic stem (ES) cells are associated with transcriptional units. Nearly half of the identified ORIs map at promoter regions and, interestingly, ORI density strongly correlates with promoter density, reflecting the coordinated organisation of replication and transcription in the mouse genome. Detailed analysis of ORI activity showed that CpG island promoter-ORIs are the most efficient ORIs in ES cells and both ORI specification and firing efficiency are maintained across cell types. Remarkably, the distribution of replication initiation sites at promoter-ORIs exactly parallels that of transcription start sites (TSS), suggesting a co-evolution of the regulatory regions driving replication and transcription. Moreover, we found that promoter-ORIs are significantly enriched in CAGE tags derived from early embryos relative to all promoters. This association implies that transcription initiation early in development sets the probability of ORI activation, unveiling a new hallmark in ORI efficiency regulation in mammalian cells.

## Author Summary

The duplication of the genetic information of a cell starts from specific sites on the chromosomes called DNA replication origins. Their number varies from a few hundred in yeast cells to several thousands in human cells, distributed along the genome at comparable distances in both systems. An important question in the field is to understand how origins of replication are specified and regulated in the mammalian genome, as neither their location nor their activity can be directly inferred from the DNA sequence. Previous studies at individual origins and, more recently, at large scale across 1% of the human genome, have revealed that most origins overlap with transcriptional regulatory elements, and specifically with gene promoters. To gain insight into the nature of the relationship between active transcription and origin specification we have combined a genomic mapping of origins at 0.4% of the mouse genome with detailed studies of activation efficiency. The data identify two types of origins with distinct regulatory properties: highly efficient origins map at CpG island-promoters and low efficient origins locate elsewhere in association with transcriptional units. We also find a remarkable parallel organisation of the replication initiation sites and transcription start sites at efficient promoter-origins that suggests a prominent role of transcription initiation in setting the efficiency of replication origin activation.

## Introduction

DNA replication initiation is thought to be the most highly regulated process in genome duplication as cells must ensure that replication origins (ORIs) fire precisely once before cell division. A large number of studies during the last twenty years have provided a good understanding of the molecular mechanisms that regulate the initiation of DNA synthesis to occur at specific chromosomal sites and during a specific window in the cell cycle to avoid undesired re- or under-replication of any part of the eukaryotic genome [1]–[3].

Less understood is how ORI specification is achieved, particularly in metazoa where ORIs are not defined by DNA sequence and the origin recognition complex (ORC) does not show sequence specificity *in vitro* [4],[5]. However, metazoan ORIs are strongly linked to other genomic functions, most notably with transcription. Transcription itself can modulate ORI activity [6]–[8], transcription factors can interact with ORC [9]–[12] and the binding of transcription factors to a plasmid can localise replication initiation to that specific site [13]. In addition, recent high-throughput studies in various experimental systems have confirmed the long observed link between early replication timing and active transcription [14]–[18]. Despite these findings, the steps in the initiation process that are influenced by transcription are poorly understood. It is possible that changes in transcriptional status could modulate the initial selection of potential ORIs either during the G1 phase of the cell cycle (pre-RC formation) or during the activation of pre-RC in S-phase.

Identification and characterisation of metazoan ORIs has been hindered by the complexity of these genomes and the lack of robust assays to comprehensively monitor DNA replication initiation. A recent genome-wide ORI mapping in HeLa cells over the regions covered by the ENCODE project has revealed that most initiation sites overlap with transcriptional regulatory elements, although there is not a direct link with gene regulation [19].

To further investigate the nature of the relationship between active transcription and ORI specification we have carried out an unbiased study of ORI location and efficiency in undifferentiated mouse embryonic stem (ES) cells. The chromatin environment of ES cells appears to be extremely permissive for gene transcription [20]. This status is maintained by hyperdynamic chromatin [21], bivalent chromatin marks [22] and Polycomb group proteins that suppress transcription at specific sites [23],[24], making the ES cell genome an excellent scenario to address the role of transcription in ORI selection and regulation. Here, we performed a high-resolution mapping of ORIs along 10.1 Mb of the mouse genome (∼0.4%) encompassing a range of genomic features characteristic of gene-rich and gene-poor regions. Replication initiation sites were identified by hybridisation of short nascent strands on tiled genomic arrays and using a stringent algorithm that takes into account the size distribution of replication intermediates relative to the initiation point. In agreement with results from human cells, we found that in mouse ES cells most of the ORIs associate with annotated transcriptional units and nearly half of them locate at promoter regions. Moreover, we found that CpG island promoter-ORIs are the most efficient ORIs in the mouse genome and that ORI specification and firing efficiency is generally maintained across cell types. The organisation of replication initiation sites at promoter-ORIs mirrors the distribution of transcription start sites (TSS) suggesting a co-evolution of the regulatory regions of replication and transcription in the genome. Interestingly, promoter-ORIs are significantly enriched in CAGE tags derived from early embryos relative to all promoters. Our findings suggest that transcription initiation early in development sets the probability of ORI firing.

## Results

### Most ORIs in the Mouse Genome Associate to Transcriptional Units

**Figure 1:**
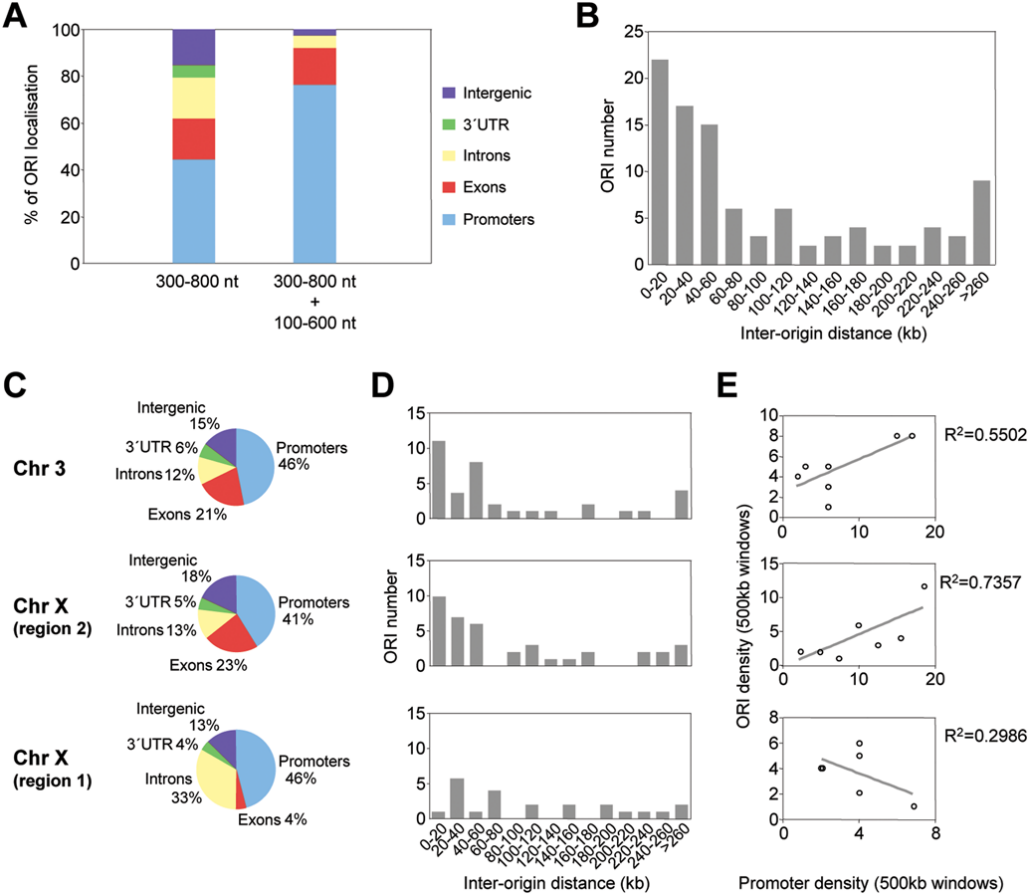
Genomic distribution of ORIs in embryonic stem cells. (A) ORI distribution at different genomic regions along 10.1 Mb of the mouse genome detected by 300–800 nt long nascent strand hybridisation (n = 97) or both by 300–800 nt and 100–600 nt long nascent strands hybridisation (n = 38). (B) Inter-origin distances along 10.1 Mb of the mouse genome (average = 103 kb; n = 97).(C–E) Distribution of ORIs and promoters in gene-rich *versus* gene-poor regions. ORI location (C), inter-origin distances (D) and density plots (E), of promoter-rich (chromosome 3 and zone 2 of chromosome X) and promoter-poor regions (zone 1 of chromosome X). The genomic features covered by the array, ORI distribution and percentages of ORI occurrence relative to the annotated genes along the 10.1 Mb and *per* region examined are summarised in Table S1.

In asynchronously growing undifferentiated mouse ES cells a large proportion of the population is in the S-phase of the cell cycle. This specific property allowed us to obtain a large enough yield in purified replication intermediates to directly hybridise genomic arrays without previous amplification (see Materials and Methods). Two biological replicates of λ-exonuclease treated short nascent strands (300–800 nt in length) were co-hybridised with genomic DNA from the same cells to tiled genomic arrays covering 10.1 Mb of the mouse genome (Agilent Technologies). Arrays were analysed by a modification of ACME (Algorithm for Capturing Microarray Enrichment) [25]. ACME identifies signals in tiled array data using a sliding window centered in each probe and returning a p-value that assesses the enrichment by comparing observed and expected number of probes above a user-specified threshold (see Materials and Methods for further details). Preparations of short nascent strands purified from asynchronously growing cells are preferentially enriched in regions close to ORIs and less enriched in their immediately adjacent sequences, showing a pine-tree distribution peaking at the ORI that allows their fine mapping by quantitative real-time PCR methods (Q-PCR) [26]–[28]. Based on this property of the nascent DNA hybridised on the arrays we filtered the results from ACME to reliably identify replication initiation sites. Windows from ACME's analysis with a p-value<0.005 were further required to have a minimum of two probes per window, an average log_2_ ratio within the window larger than the 75^th^ percentile of the data, and the defining probe of a window above the threshold and with a p-value<0.005 (see Materials and Methods). Replicate experiments showed a high degree of correlation and were averaged (R^2^ values of 0.954). Applying this stringent algorithm we identified 97 ORIs that mostly map associated to annotated transcriptional units (85%) and, specifically, at promoter regions (44%, from which 88% correspond to CpG island-promoters) (Table S1 and Figure 1A, left column).

Replication initiation at CpG islands in mammalian cells is well documented [29],[30] and our method identifies the ORIs associated with the CpG islands of the *Hprt1* and *Mecp2* genes precisely at the previously described sites, validating the quality of our ORI maps (ORIs 45236 and 67276, Table S2) [31],[32]. Our criterion detects ORI activity at 32% of all known promoters covered by the array (50% of the annotated CpG islands and 8% of the annotated non-CpG island promoters, Table S1). This result highlights at genomic scale the link between the regions that trigger replication and transcription initiation that has been previously suggested in studies at specific loci [26], [27], [29], [33]–[35].

**Figure 2:**
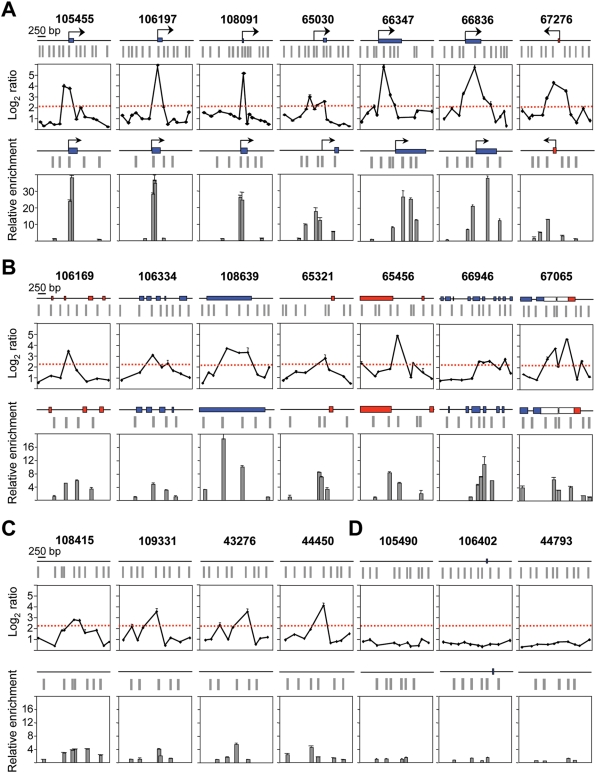
Sensitivity of the ORI identification method. Array profiles and nascent strand abundance measurements by Q-PCR of 18 positive regions located at 5′ends of genes (A), at less than 200 bp of exons (B), including one at the 3′ UTR of two genes of convergent transcription (ORI 67065), or at intergenic zones (C). Similar analysis was performed for 3 negative regions (D). The maps above each graph show the annotated genomic features and probe distribution of the regions analysed. Blue and red rectangles indicate exons transcribed from the upper or the lower strand, respectively, and black arrows show the position of the major annotated TSS. Grey rectangles represent array probes. The red dashed line depicts the threshold of the array duplicates. Q-PCR experiments were carried out in duplicate in at least two independent preparations of 300–800 nt long nascent strands and values were normalised to the flanking primer pair detecting the lowest amount of nascent strands at each region. Standard deviation bars are indicated. Primer pairs were designed to amplify across the array probes in all possible cases and their sequences are shown in Table S3. ORI 67276 corresponds to the CpG island region of the *Mecp2* gene.

Our results increase by more than one order of magnitude the number of characterised ORIs in the mouse genome. In addition, the small length of the nascent strands hybridised on the arrays and the window size chosen for the analysis allowed us to accurately define replication initiation sites within an 800 bp region (Table S2 and Figures 2–4).

**Figure 3 pgen-1000446-g003:**
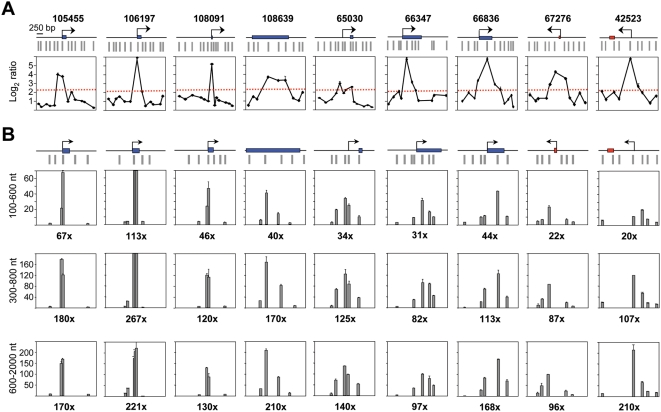
Replication initiation activity at CpG island-ORIs. (A) Array profiles of 9 CpG island-ORIs. Symbols are like in Figure 2.(B) Q-PCR measurements of nascent strands abundance across the positive probes defining the ORIs shown in A in preparations of replication intermediates of the indicated sizes. Primer pairs span less than 2 kb at each region and values were normalised to the average of those obtained at the three negative regions in each gradient fraction. Numbers below each panel indicate fold enrichment of the ORI peak relative to the averaged negative regions. Primer pair sequences are listed in Table S3.

**Figure 4:**
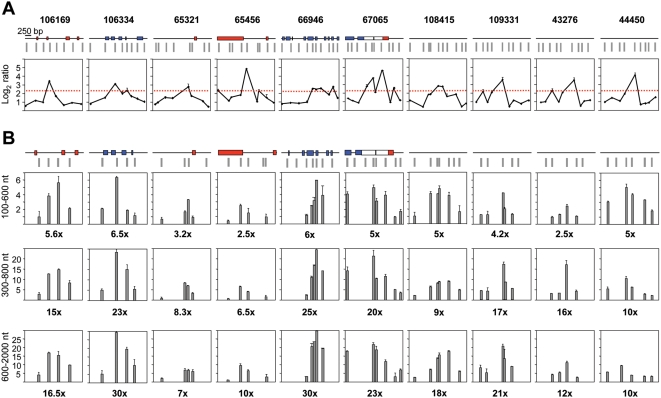
Replication initiation activity at non-promoter-ORIs. Same analysis as on Figure 3 for 10 non promoter-ORI regions.

The identified ORIs were distributed at an average interorigin distance of 103 kb, however, half of them map within 60 kb distance suggesting a degree of ORI clustering (Figure 1B). To test whether this distribution was related to gene organisation, we analysed separately gene rich regions (3.2 Mb on chromosome 3 and 4 Mb at region 2 of chromosome X) and gene-poor regions (2.9 Mb at region 1 of chromosome X) (Table S1). At both gene-rich regions, ORI localisation and interorigin distances were comparable and ORI density positively correlated with promoter density (Figure 1C, 1D and 1E, upper two graphs). By contrast, at the gene-poor region 1 of chromosome X we found no ORI clustering and no correlation between promoter density and ORI density, although the percentage of ORIs associated with promoters was similar at the three regions analysed (Figure 1C, 1D, and 1E, lower graphs). These differences in ORI density were due to non promoter-ORIs being very sparsely distributed along gene-desert regions and suggest a coordinated organisation of replication and transcription in the mouse genome, in line with conclusions reached by genome-wide studies in other systems [16],[19].

### CpG island-ORIs Are the Most Efficient ORIs in Embryonic Stem Cells

To validate our algorithm for ORI identification we selected 18 positive and 3 negative regions and analysed their abundance in independent preparations of purified 300–800 nt nascent strands by Q-PCR. Since Q-PCR defines ORIs as regions preferentially amplified in relation to their flanking sequences, we interrogated each region with 4 to 6 primer pairs spanning 2 kb across the probes defining the ORI and normalised the values to the flanking pair detecting the lowest amount of nascent strands in each case. The regions studied were representative of the observed ORI location relative to the genes. The average log_2_ ratios of the array duplicates for each region are shown in the top panels of the figure below the corresponding genomic maps (Figure 2). Seven of these mapped at CpG island promoters (including the ORI previously identified at the *Mecp2* CpG island, ORI 67276, Figure 2A) [32], six mapped at or immediately adjacent to exons, one mapped at the 3′UTR of two genes with convergent transcription (Figure 2B), and four mapped to intergenic regions (Figure 2C). In all 18 regions, the significant probes identified on the arrays coincided with the point of higher enrichment in nascent strands relative to its immediate flanking sequences by Q-PCR. Detected enrichments ranged between 16 to 40 times at CpG island-ORIs (Figure 2A) and between 4 and 10 times at non promoter-ORIs (Figure 2B and 2C). An interesting exception was ORI 108639 (Figure 2B, third panel) that maps at the last exon of the *Zfp697* gene and was 18 times enriched in nascent strands relative to its local flank. This region harbours a C+G composition and CpG density that qualifies it as a 3′ CpG island, suggesting that this ORI could map at an unannotated promoter (see below). In contrast, the regions that were scored as negative by our algorithm showed no enrichment in nascent strands by Q-PCR (Figure 2D), indicating that even low efficiency replication initiation sites detected in the arrays were indeed ORIs. It is worth noting that the peaks defined by the nascent strand profiles were in all cases within 800 bp width, coinciding with the upper size of the replication intermediates hybridised in the arrays and constituting the highest resolution genomic mapping of mammalian ORIs to date.

Q-PCR results suggested that CpG island promoter-ORIs were generally more efficient than non promoter-ORIs. As the arrays were hybridised with non-amplified short nascent strands, the output log_2_ ratios should give semi-quantitative information about ORI efficiency. Consistently, the hybridisation signals obtained at the CpG island-ORI class (mean values of 3.899) were significantly higher (*p* = 0.00005, Welch Two Sample T-test) than those at the non promoter-ORI class (mean values of 3.008). To be able to compare ORI efficiencies directly, we performed Q-PCR on three consecutive sucrose gradient fractions containing nascent strands of 100–600, 300–800 and 600–2000 nt in length, respectively, and normalised the abundance relative to that obtained at the negative regions in each gradient fraction (Figures 3 and 4). We found that the maximum enrichment in replication intermediates detected by Q-PCR coincided with the highest point of the log_2_ ratio profile in all gradient fractions (Figures 3A and 4A), confirming that positive regions identified in the arrays were genuine ORIs. In addition, ORI enrichment relative to the non-ORI regions increased with the size of the nascent strands analysed (fold enrichment of the ORI peak relative to the negative regions are indicated below each histogram), further supporting that DNA synthesis elongates from these regions to replicate the genome (Figures 3B and 4B). Remarkably, nascent strand enrichments detected at CpG island-ORIs (Figure 3B) were one order of magnitude higher than those detected at non promoter-ORIs across all gradient fractions (Figure 4B), implying that CpG island-ORIs are the subset of ORIs that are preferentially activated in the analysed cell population.

Preparations of 100–600 nt nascent strands likely contained Okazaki fragments that co-purified with these small replication intermediates. Given that asynchronously growing ES cells were used, Okazaki fragments were expected to derive from the entire genome and to diminish the overall level of enrichment without a bias for any particular loci. Increasing the background signal, however, could critically affect the detection of weak ORIs, as seen at most non promoter-ORIs in the 100–600 nt nascent strand fraction (Figure 4B). We reasoned that only the ORIs that are active in the majority of cells of the analysed population would be enriched enough in small size nascent strand preparations to give a significant signal on the arrays and could, therefore, identify a collection of the most efficient ORIs. To test this possibility, we hybridised two more arrays with preparations of shorter nascent strands (100–600 nt in length) derived from the same cells. When considering the data from the four arrays altogether, the number of identified ORIs dropped from 97 to 38 and, interestingly, their genomic distribution changed dramatically (Figure 1A, right column and Table S2). In this case, more than 97% of the identified ORIs mapped at transcriptional units and 78% at promoter regions (of those, 96% correspond to CpG island promoters), indicating that CpG island promoter-ORIs are the subset of ORIs that fire with higher efficiency in mouse ES cells. A similar conclusion can be reached when examining the distribution of the 97 identified ORIs in gene poor versus gene rich regions (Table S1). While the proportion of mapped ORIs associated with CpG islands was similar in both cases (38% *vs* 44 and 36%), the proportion of annotated CpG island-promoters showing ORI activity at gene poor versus gene rich regions was 75% *vs* 54 and 45%, respectively (Figure 1C and Table S1).

It should be noted that our experimental approach for ORI identification is not suited to detect ORIs dispersed across large regions, such as the ORI downstream of the DHFR gene in hamster CHO cells [36]. We could not, therefore, address either the abundance of broad initiation regions in the genome nor their firing efficiency.

### ORI Firing Efficiency Is Maintained across Cell Types

**Figure 5:**
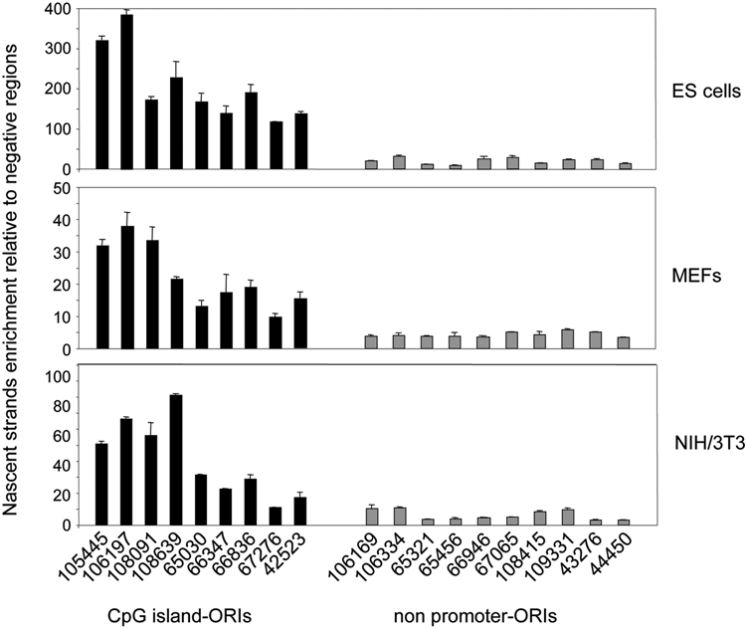
ORI specification and firing efficiency across cell types. Relative abundance of 9 CpG island-ORI regions and 10 non promoter-ORI regions in 300–800 nt long nascent strands derived from ES cells, MEFs and NIH/3T3 fibroblasts. Averaged values for the non-ORI regions were considered as baseline in each cell type.

**Figure 6:**
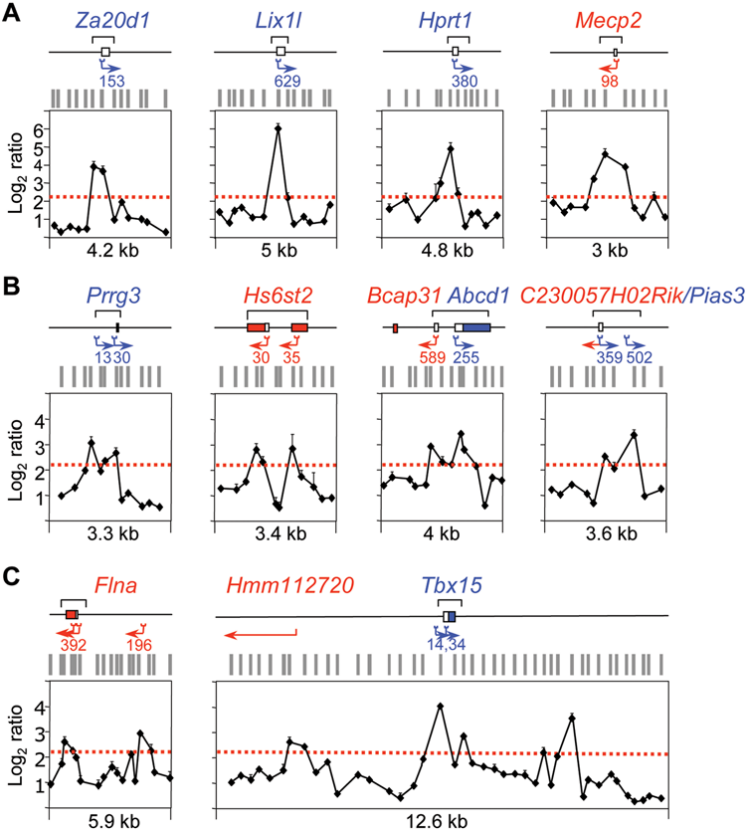
Organisation of replication and transcription initiation at promoter-ORIs. Maps indicate the number and position of the CAGE tags annotated at CpG island promoter-ORIs with unidirectional (A) and with alternative or bidirectional transcriptional activity (B) [37]. Blue and red arrows indicate transcription from the upper or the lower strand, respectively, and brackets show the position of the CpG islands. Graphs show the nascent strand profiles of the arrays hybridised with 300–800 nt preparations along the same regions. Other symbols are like in Figure 2. (C) Analysis of the TSS and nascent strand profiles at the *Flna* and *Tbx15* loci.

To check whether this difference in ORI usage was conserved in other cell types we studied ORI firing efficiency by Q-PCR at 9 CpG island-ORIs and 10 non promoter-ORIs in preparations of 300–800 nt long nascent strands derived from mouse embryonic fibroblasts (MEFs) and NIH/3T3 transformed fibroblasts. We first analysed if DNA replication initiated at these sites in differentiated cell types by scanning a 2 kb region surrounding the ORI in experiments analogous to those shown in Figures 3 and 4 (Figure S1). Overall enrichments in replication intermediates detected at ORI regions relative to the negative zones were smaller in MEFs and 3T3 fibroblasts compared to ES cells, likely reflecting the differences in cell division rates between the three cell types. Despite this, the analysed regions showed peaks of enrichment relative to flanking sequences and to the non-ORI regions at the same positions observed in ES cells, suggesting that ORI specification is maintained at these sites across the three cell types. Although relative activity varies between ORIs in the three cell lines, two clearly distinct groups of ORIs can be distinguished on the basis of their efficiency (Figure 5). Strong ORIs correspond to CpG island ORIs (black histograms), while weak ORIs correspond to non promoter-ORIs (grey histograms). Interestingly, the replication initiation activity of the CpG island associated to the last exon of the *Zfp697* gene (ORI 108639) was indistinguishable from CpG island promoter-ORIs in the three cell types studied, suggesting that CpG regions might contain a hallmark for efficient replication initiation regardless of their position relative to the gene. An exception to this general tendency is ORI 67276 (*MeCp2* CpG island), the weakest CpG island-ORI even in ES cells. This is possibly due to the fact that the primer pairs detecting the highest enrichment in nascent strands were not adjacent to the TSS underestimating the real activity of this ORI (Figures 3 and 6A, and see below). Altogether, these results indicate that ORI prominence is generally retained from pluripotent cells to differentiated cells and cell lines.

It is important to note that the higher efficiency in ORI activity found at CpG islands is not due to the overreplication occurring at promoter-ORIs that we recently reported [33]. Overreplicated intermediates are typically 100–200 bp long and their detection strictly relies on the use of cloned DNA to normalise primer pair efficiency. In this work we consistently normalised the data with genomic DNA that suppresses all possible contribution of overreplicated short fragments (either for array hybridisations or for Q-PCR measurements, see Materials and Methods). In addition, ORI firing efficiency at CpG islands was found to be consistently higher than at non-promoter ORIs along nascent strand preparations of increasing sizes, where the contribution of short overreplicated fragments is negligible (Figure 3B).

### Highly Efficient ORIs Are Strictly Associated with TSS

Closer examination of the log_2_ ratio profiles across several CpG island regions similar to those shown in Figure 3 indicated that maximum enrichments in short replication intermediates were usually detected around the major transcription initiation site annotated at each promoter (mouse NCBI database build 36.1). We investigated this correlation in more detail taking advantage of the accuracy of our ORI mapping and the recently available high coverage annotation of transcription start sites (TSS) derived from 145 mouse libraries by extensive CAGE and PET analysis (http://gerg01.gsc.riken.jp/cage/mm5) [37]. Figure 6 shows several examples of CpG island promoter-ORIs with unique or multiple TSS displaying the number and position of annotated tags defining each TSS alongside our array results. The replication initiation points defined by the log_2_ ratios exactly parallel the transcription initiation sites defined by tag sequencing at less than 30 bp resolution in most cases (Figure 6A). This correlation was more striking in the case of CpG island-promoters with alternative transcription initiation sites or bidirectional activity where, when array probe distribution allowed it, distinct replication initiation points located immediately adjacent to the mapped tags at those regions could be clearly distinguished (Figure 6B).

**Figure 7:**
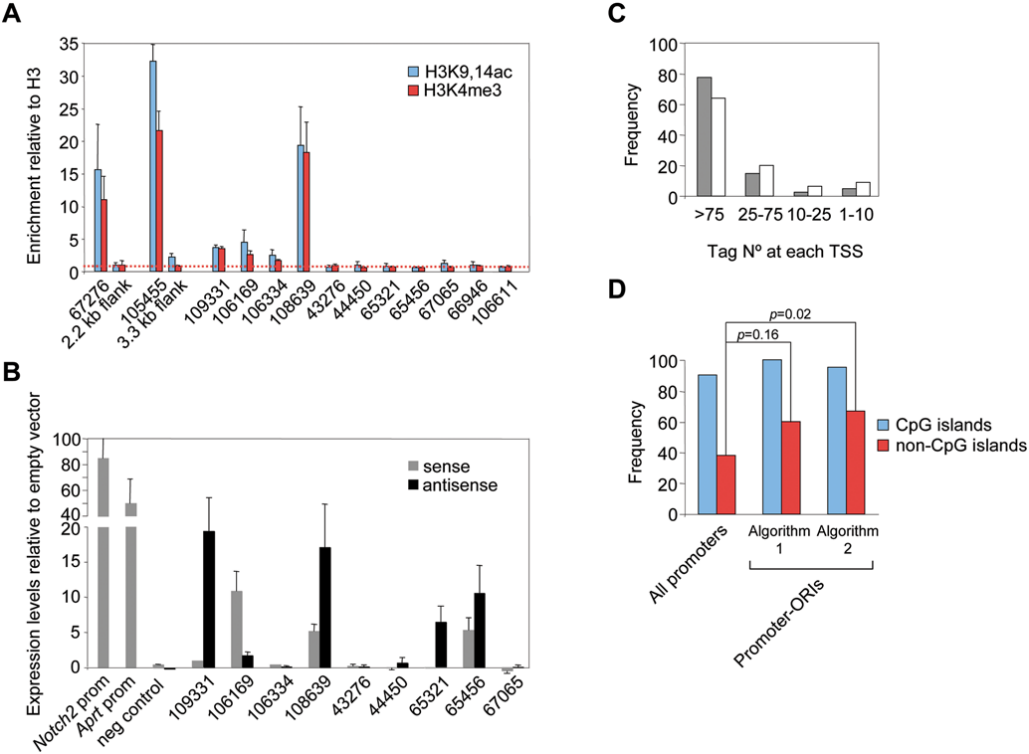
Prediction of novel TSS and association with embryonic transcription. (A) Enrichment for H3K4me3 and H3K9,14ac modifications relative to total H3 detected by ChIP. Values for the regions flanking *Mecp2* and *Zad20d1* CpG island-ORIs (ORIs 67276 and 105455, respectively) were considered as baseline. Q-PCR reactions were carried out in duplicate in three independent preparations of immunoprecipitated material. Standard deviation bars are indicated. (B) Expression levels relative to empty vector in transient transfection reporter assays. Constructs carrying the *Notch2* and *Aprt* promoters cloned in the sense orientation were used as positive controls and a region at the first intron of the *Notch2* gene cloned in both orientations as the negative one. Histograms represent the averaged normalised values of two independent transfections carried out in duplicate. Standard deviation bars are indicated. Primer pair sequences used and the sizes of the cloned fragments are listed in Table S3. (C) Frequency of promoter-ORIs relative to the number of mapped CAGE tags at each TSS [37]. Grey bars represent ORIs identified by the strict algorithm (n = 40) and white bars ORIs identified when applying a less stringent algorithm (n = 75). (D) Frequency of total promoters or promoter-ORIs transcriptionally active in early development [37]. A chi-square test was used to compare the frequency of tagged promoters between promoter-ORIs identified by the algorithms and the rest of promoters.

Based on these observations, we asked whether two independent clusters of TSS for the same gene, but not located within the same CpG island region, were also associated with replication initiation sites. We analysed the *Flna* gene, which is transcribed from two alternative promoters, one located in a CpG island and another one 3.4 kb upstream that is not CpG island-associated. Our algorithm identified two separated peaks of nascent strands enrichment pointing exactly to the two tag clusters from where the transcription of the gene initiates (Figure 6C, left graph). Then we asked the reciprocal question, can alternative or novel TSS be identified from the nascent strand profiles? To test this possibility we analysed 12.6 kb surrounding the CpG island associated with the *Tbx15* gene, where three distinct ORIs were identified in our arrays (Figure 6C, right graph). As anticipated, the peak located at the CpG island pointed to the mapped tags for that gene. The peak located 4 kb upstream marked exactly the predicted 5′ position of the transcript of a model gene, *Hmm112720*, and the peak located 3.5 kb downstream of the *Tbx15* ATG initiation codon was orphan in terms of transcription initiation activity. To check whether this ORI was associated with an uncharacterised TSS we analysed the chemical modifications of the histone components of the resident nucleosomes by chromatin immunoprecipitation (ChIP) and its capacity to drive transcription in plasmid reporter assays (Figure 7A and 7B, respectively). We found that the nucleosomes at this ORI were enriched in histone H3 lysine 4 trimethyl (H3K4me3) and histone H3 lysine 9 and 14 acetyl (H3K9,14ac) marks, characteristic of transcription initiation (ORI 109331, Figure 7A) [38]. The enrichment in both histone modifications detected at ORI 109331 was significantly lower compared to that detected at CpG island promoter-ORIs, consistent with H3K4me3 levels being positively correlated to gene expression rates and therefore indicating that this TSS might correspond to a low transcribed RNA [39]. In agreement with this, a 944 bp fragment containing ORI 109331 displayed promoter activity in reporter assays in the antisense orientation, although less efficiently than canonical CpG island promoters (*Notch2* and *Aprt* promoters, Figure 7B). Our results help to explain previous observations of ORI clustering in CG-rich regions [19],[40], that most likely correspond to discrete replication initiation sites associated to distinct TSS that are activated in different cells of the analysed population.

The above results demonstrate a strong correlation between the initiation of replication and transcription at CpG island promoters and at clustered ORIs at promoter-rich regions. To address how general this association was and to test whether non-promoter ORIs might be good predictors of novel TSS regardless of their location in the genome, we extended the analysis of the histone signatures by ChIP to another 10 randomly selected non-promoter ORI regions. Figure 7A shows the proportion of H3K4me3 and H3K9,14ac modifications relative to total H3 detected at the studied regions in comparison with that detected at *Mecp2* and *Zad20d1* CpG island-ORIs and their flanks (ORIs 67276 and 105455). Three out of the ten regions tested (ORIs 106169, 106334 and 108639) were enriched in both histone modifications relative to background and the negative controls, indicating that these ORIs could be linked to transcription initiation [39],[41]. Consistently, ORIs 106169 and 108639 also displayed promoter activity in reporter assays, as well as another two ORIs that were not enriched in H3K4me3 or H3K9,14ac histone marks (ORIs 65321 and 65456, Figure 7B). As 44% of the ORIs identified in this study were located at well characterised promoters, altogether these data suggest that a minimum estimate of 60% of the ORIs in the genome of mouse ES cells are associated to TSS.

### Transcription Initiation in the Embryo Specifies Replication Origin Efficiency

The data presented in Figure 6 shows that ORI architecture at CpG island regions is reminiscent of that of promoters, where discrete initiation sites can be distinguished, each of them mapping immediately adjacent to annotated TSS. This similar organisation of the regions driving replication and transcription initiation, together with the finding that CpG islands are the most efficient ORIs in the genome, suggests that both processes might benefit from each other. This hypothesis makes several testable predictions that we evaluate in turn.

First, highly efficient ORIs would be expected to be preferentially associated to promoters driving ubiquitous expression. We considered the number of tags mapped at the TSS of each promoter-ORI as an indicator of relative promoter usage across several tissues and cell types [37] and analysed them relative to ORI occurrence. As expected, 78% of the ORIs locate at TSS where more than 75 clustered tags have been identified, representing the promoters of the most widely expressed genes at the studied regions (Figure 7C, grey histograms).

Second, many promoters in the genome should display replication initiation activity. To test this possibility we reanalysed our array data using a less stringent algorithm (see Materials and Methods). The strict algorithm detected ORI activity at 32% of annotated promoters (Table S1); applying the less stringent criterion we now detected ORI activity at 60% of the known promoters (83% of annotated CpG islands and 33% of the annotated non-CpG island promoters) (Table S1). Although in this case the association is slightly less prominent, ORIs also occur with higher frequency at the promoters with a higher number of mapped tags (Figure 7C, white histograms).

Finally, if the spatial coincidence between replication and transcription initiation sites has a functional significance we would predict that promoter-ORIs would be transcriptionaly active in early development. To test this hypothesis we again surveyed the mouse CAGE database and analysed the tags derived from embryonic or germ line libraries mapped at each promoter-ORI relative to all known promoters [37]. CpG island associated genes, including those of tissue specific expression, are transcribed in the germ line [42]–[45], and we consistently found tags derived from early embryos and testis libraries at 90% of the CpG islands present in the array. When we performed similar analysis for CpG island-ORIs, the proportion increased to 100 and 95% (Figure 7B, blue histograms, first and second algorithm, respectively). More strikingly, we found CAGE tags derived from early embryos at 60 and 67% of non-CpG island promoter-ORIs, which shows statistical evidence of enrichment as the observed frequency of expression of this type of promoters at early developmental stages is only 38% (Figure 7D, red histograms, first and second algorithm, respectively). Interestingly, the ORI 108639, located at a 3′ CpG island spanning the last exon of the gene *Zfp697* that is highly active in all cell types analysed (Figure 5), has also associated tags derived from early embryos. These results strongly indicate that the most efficient ORIs in the genome are those associated with sites of embryonic transcription initiation.

## Discussion

By combining a genome-wide approach to identify preferential sites of DNA replication initiation with in depth analysis at distinct classes of ORIs according to their genomic location, we were able to conclude that ORI firing efficiency is strongly associated to transcription initiation activity. The short size of nascent strands hybridised in the arrays and the stringent algorithm chosen to analyse the datasets allowed us to draw a highly accurate map of 97 new ORIs along 10.1 Mb of the mouse ES genome. A systematic analysis of the location of the identified ORIs revealed a strong correlation with annotated transcriptional units and specifically with the annotated 5′ ends of genes (Figure 1). This genomic distribution of ORIs is similar to that reported in a recent analogous study in HeLa cells [19], a remarkable fact given the diverse genetic and epigenetic status of both cell types and the differences in the technical approaches used to prepare the nascent strands for array hybridisations. Therefore, these two high-resolution ORI maps likely represent a comprehensive picture of the coordinated organisation of replication and transcription in the mammalian genome. However, a significant fraction of the ORIs identified in both experimental systems are not associated with known promoters nor carry the histone modifications indicative of transcriptional activity, implying that ORI specification can be achieved by several mechanisms.

Detailed measurements of nascent strand abundance at both classes of ORIs in preparations of replication intermediates of increasing sizes (Figures 3 and 4), and array hybridisation with very short nascent strands that mainly represent highly active ORIs (Figure 1A and Table S2), indicated that the most efficient replication initiation sites are those associated with CpG island promoters. Interestingly, this hierarchy of ORI usage not only occurs in mouse ES cells but is also maintained in differentiated cells and cell lines (Figure 5), suggesting that firing efficiency is linked to transcription initiation activity. Recently it has been reported that more than half of all mouse and human genes are associated with TSS driving divergent transcription over short distances, proposed to help maintain promoter regions in a state poised for subsequent regulation [46],[47]. We did not find any preferential representation of this class of promoters at the promoter-ORI class, suggesting that neither ORI specification nor activity are linked to this type of transcriptional regulation.

Our results support the Jesuit model of ORI initiation proposed in the late 90's by Melvin DePamphilis (“many are called, but few are chosen”) [48],[49]. According to this model, the metazoan genome contains multiple potential sites of replication initiation whose activity is modulated during the G1 phase of each cell cycle by a combination of parameters such as nuclear organisation, chromatin structure, gene transcription or DNA sequence. This study identifies transcription initiation early in development as a strong determinant of ORI efficiency in mammalian cells. Transcription start sites of active genes are usually nucleosome-free indicating a more open chromatin conformation [38],[39] and presumably the parasitism of ORIs at TSS would increase the chances of firing through the facilitation of the assembly of the replication complexes to these sites. Indeed, a recent report showed that ORC binding to the Epstein-Barr virus origin of plasmid replication is stabilised by RNA [50], opening the possibility that nascent RNA molecules could contribute to ORC recruitment in mammalian cells. Interestingly, we found that ORI and promoter organisation are virtually identical (Figure 6), likely reflecting that the initiation of replication and transcription are influenced by the same chromatin constraints. Moreover, we were able to show that the probability of ORI activation is set by transcription initiation early in development: we found that promoter-ORIs are significantly enriched in CAGE tags derived from early embryos relative to the rest of promoters (Figure 7D) [37].

Our results point to a scenario where active promoters in germ cells and early embryonic cells will recruit pre-RCs and acquire the capability to drive replication. It is possible that the initiation of both replication and transcription at these promoter-ORIs will contribute to the configuration of a competent chromatin conformation that is a prerequisite for efficient replication initiation. This epigenetic state would then be transmitted and maintained in somatic cells. The above scenario can accommodate several observations made in various developmental systems. For example, in somatic cells, silent CpG islands on the inactive X chromosome function as ORIs as efficiently as their counterparts on the active X [32]. On the other hand, upon activation at specific developmental stages new ORIs are switched on while others are maintained [51],[52].

In addition, our work could provide experimental evidence in support of a hypothesis for the origin of CpG islands [53]. These authors proposed that CpG islands have acquired their distinct properties of C+G composition, CpG density and lack of DNA methylation due to their dual role as promoters and ORIs early in development. Since the number of CpG island associated genes is significantly smaller in mouse than in humans [54]–[56], presumably due to the different rates of CpG loss occurring during mammalian evolution [57],[58], we hypothesise that promoter-ORIs showing early embryonic expression that are not linked to CpG islands in the mouse genome would be CpG island associated in the human genome. To test this possibility we thoroughly searched for the presence of CpG islands at the human orthologous regions of the mouse promoter-ORIs identified in our work (human NCBI database build 36.3). We found that 50% of the non-CpG island associated promoter-ORIs expressed early in mouse development indeed harbour a CpG island in the human genome. However, the observed frequency of this association when considering all other promoters is only 10% (p-value = 0.007), making it tempting to speculate that the co-evolution of the regulatory regions driving replication and transcription initiation could have contributed to the shape of the mammalian genome.

## Materials and Methods

### Cell Culture

The mouse embryonic stem cell line PGK12.1 was grown as described [59]. Mouse embryonic fibroblasts (MEFs) were derived from 12.5 dpc CD1 embryos and grown in F12 Nutrient Mixture (Ham) medium supplemented with 10% FCS, 1×10^5^ U/ml penicillin, 100 mg/ml streptomycin, 2 mM L-glutamine, 1× non-essential amino acids, and 50 µM β-mercaptoethanol (Invitrogen). NIH/3T3 cells were cultivated as recommended in the ATCC.

### Nascent Strand Purification

Genomic DNA isolation and nascent strands fractionation was performed as described [33]. Sucrose gradient fractions containing replication intermediates ranging between 100–600 nt, 300–800 nt and 600–2000 nt in size were subjected to digestion by λ-exonuclease, which degrades contaminating random sheared DNA leaving untouched DNA replication intermediates that are protected by a 5′ RNA-primer, as described [28]. ORI enrichment in 300–800 nt nascent strand preparations was routinely monitored by Q-PCR for *Mecp2* CpG island-ORI region [32] and a flanking region located 1 kb downstream as control. Primer sequences are provided in Table S3. Only preparations showing a minimum of 5-times enrichment were used in array hybridisation experiments or Q-PCR validations. Three to four µg of λ-exonuclease treated nascent strands purified from 5×10^9^ mouse embryonic stem cells were co-hybridised with the same amount of total genomic DNA for each array replicate.

### Hybridisation of DNA Microarrays

Sample labelling, hybridisation and data extraction were performed according to standard procedures from Agilent Technologies (2005). Agilent 22K feature arrays were designed to cover two 4 Mb regions on the X chromosome (45.5–49.5 Mb and 65–69 Mb) and a 4 Mb region on chromosome 3 (95.5–99.5 Mb) of non-repetitive DNA sequences, with an average coverage of one 60-mer probe each 250 bp (Oxford Gene Technology). Probe design was based on Ensembl mouse build 35.

### Data Normalisation and Analysis

Raw datasets from each experiment were loess normalised to remove signal intensity-dependent bias using GeneSpringX software (Agilent). Normalised data were analysed with the ACME algorithm [25], that uses the following approach to examine enrichment. First, using a user-specified threshold (0.95 in our case) probes are divided into positive probes (those with a log_2_ ratio larger than the specified quantile) and negative ones. ACME then uses a sliding window of fixed size (800 bp in our case) centered on each probe. Within each window, a chi-square test is used to examine enrichment by comparing the observed number of positive probes with the expected number. The p-value can be used as a rough guide to determine regions of interest (in our case, we used as cut-point a p-value<0.005). The original results from ACME are referred to as “Algorithm 2” in Figure 6, and “the less stringent algorithm” in the text. We further filtered the regions identified with ACME as follows: (i) regions were required to contain at least two probes, (ii) the average log_2_ ratio within a window had to be larger than the 75^th^ percentile of the data and (iii) the defining probe of a window had to be above the threshold and have a p-value<0.005. These additional conditions were used to minimise false positives by excluding single-probe windows, requiring all of the probes within a region to show at least some evidence of enrichment, and preventing a window from being labelled as interesting simply because of it being next to a highly enriched window. The final list of significant probes defining each 800 bp window is shown in Table S2. These filtered results are what we refer to as “Algorithm 1” in Figure 6, and “the more stringent algorithm” in the text. ACME analyses were carried out using R [60] and the BioConductor package ACME [25]. All data have been deposited in the Gene Expression Omnibus (GEO), http://www.ncbi.nlm.nih.gov/geo/query/acc.cgi?accGSE15082.

Replication intermediates abundance relative to annotated genomic features was first analysed by visual inspection using the GEB browser (http://web.bioinformatics.ic.ac.uk/geb) and manually validated in the mouse NCBI database build 36.1. Annotations for genes and transcripts were obtained from RefSeq, Ensembl and UniGene databases. CpG islands were identified using the strict algorithm displayed in the NCBI database: minimum length of 500 bp, minimum C+G content of 50% and minimum observed CpG/expected CpG of 0.6 [61].

### Quantitative Real-Time PCR

Quantitative real-time PCR was performed with an ABI Prism 7000 Detection System (Applied Biosystems), using SYBR Premix Ex Taq (Takara Bio Inc.) and following manufacturer's instructions. Four serial 10-fold dilutions of sonicated genomic DNA were amplified using the same reaction mixture as the samples to construct the standard curves. Primer sequences are indicated in Table S3. All real-time PCR reactions were performed in duplicate and in at least two independent preparations of nascent strands or immunoprecipitated material. Quantitative analyses were carried out using the ABI Prism 7000 SDS Software (version 1.2.3).

### Chromatin Immunoprecipitation

PGK12.1 cells cross-linking and chromatin immunoprecipitations were performed as described [62], with the following modifications. Cells were harvested in Lysis Buffer I (5 mM PIPES pH 8.0, 85 mM KCl, 0.5% NP-40, and protease inhibitors). Nuclei were pelleted by centrifugation, resuspended in Lysis Buffer II (50 mM Tris pH 8.0, 1% SDS, and 10 mM EDTA pH 8.0, and protease inhibitors) and disrupted by sonication using Bioruptor (Diagenode), yielding genomic DNA fragments with a size distribution of 100–800 bp. For each ChIP 25 µg of chromatin were immunoprecipitated with the following polyclonal antibodies: H3 acetyl K9,14 (5 µg, Upstate), H3 tri methyl K4 (2 µg, Abcam), or H3 (2 µg, Abcam). Immune complexes were recovered by the addition of 20 µL of blocked protein A/G Plus beads (Santa Cruz) and washed and eluted as described [62].

### Luciferase Reporter Assays

PCR-amplified DNA fragments were cloned in both orientations upstream of the luciferase gene in the pGL3 basic vector (Promega). Constructs were cotransfected with a *Renilla* Luciferase Control Reporter Vector (pRL-SV40, Promega) using Lipofectamine 2000 (Invitrogen) and following manufacturer's instructions. Firefly and *Renilla* luciferase signals were quantified 30 h post-transfection using the Dual-Luciferase Reporter Assay System (Promega). Reporter expression was normalised with the *Renilla* luciferase signal and averaged across two independent transfections carried out in duplicate. Primer sequences used to amplify the fragments for cloning and insert sizes are provided in Table S3.

## Supporting Information

Figure S1 Replication initiation activity at CpG island-ORIs and non promoter-ORIs in MEFs and NIH/3T3 cells. (A) Q-PCR measurements of nascent strands abundance across the positive probes defining the ORIs identified in ES cells in preparations of replication intermediates of 300–800 nt derived from MEFs. Normalisations were as in Figure 3. (B) Equivalent analysis as in A for preparations of replication intermediates of 300–800 nt derived from NIH/3T3 cells.
(2.91 MB TIF)

Table S1 Summary of the ORI mapping data. Genomic features covered by the array, ORI distribution and percentages of ORI occurrence relative to the annotated genes along the 10.1 Mb and *per* region.
(0.08 MB DOC)

Table S2 List of the 97 newly identified mouse ORIs. The sequence of the 60-mer probe centred at the 800 bp significant window, the starting and ending position of that probe in the Ensembl mouse build 35 and the location relative to the genes is indicated. Blue rows show the 38 ORIs that were also identified in the arrays hybridised with 100–600 nt long nascent strands.
(0.12 MB XLS)

Table S3 List of the primers used in this work.
(0.32 MB DOC)
