# Supplementary figures and images for: Transcription Initiation Activity Sets Replication Origin Efficiency in Mammalian Cells
Source: PLoS Genet. 2009 Apr 10;5(4):e1000446. doi: 10.1371/journal.pgen.1000446 (PMC2661365; doi:10.1371/journal.pgen.1000446)

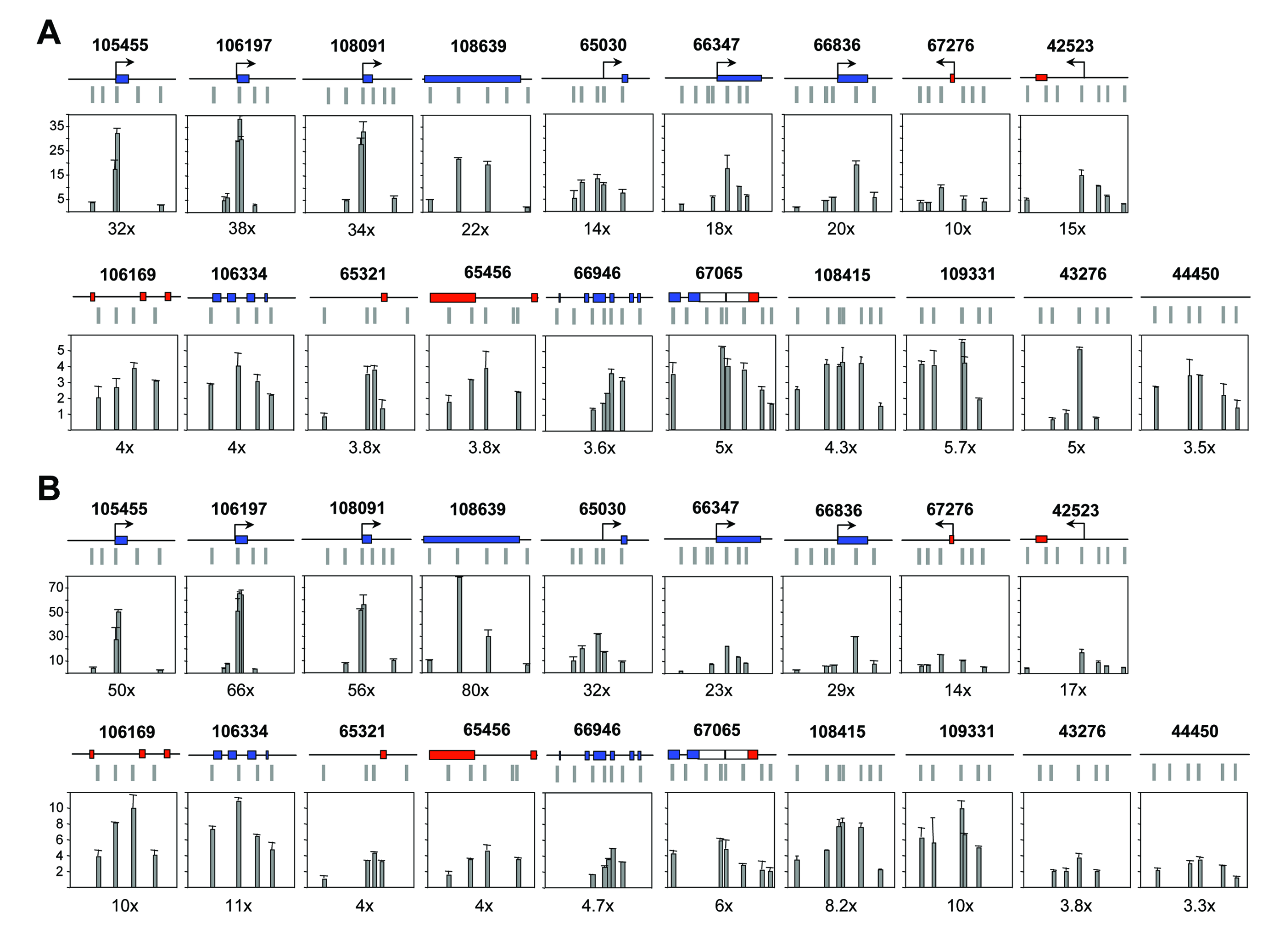

Supplement: Figure S1 — Replication initiation activity at CpG island-ORIs and non promoter-ORIs in MEFs and NIH/3T3 cells. (A) Q-PCR measurements of nascent strands abundance across the positive probes defining the ORIs identified in ES cells in preparations of replication intermediates of 300–800 nt derived from MEFs. Normalisations were as in Figure 3. (B) Equivalent analysis as in A for preparations of replication intermediates of 300–800 nt derived from NIH/3T3 cells. (2.91 MB TIF) [file pgen.1000446.s001.tif]
