# Supplementary material for: Transcription Initiation Activity Sets Replication Origin Efficiency in Mammalian Cells
Source: PLoS Genet. 2009 Apr 10;5(4):e1000446. doi: 10.1371/journal.pgen.1000446 (PMC2661365; doi:10.1371/journal.pgen.1000446)
Supplement: Table S1 — Summary of the ORI mapping data. Genomic features covered by the array, ORI distribution and percentages of ORI occurrence relative to the annotated genes along the 10.1 Mb and per region. (0.08 MB DOC) [file pgen.1000446.s002.doc]

|  | | **CHROMOSOME 3** | **CHROMOSOME X, region 1** | **CHROMOSOME X, region 2** | **TOTAL** |
| --- | --- | --- | --- | --- | --- |
| **ARRAY**  **CHARACTERISTICS** | Size (bp) | 3243959 bp | 2885381 bp | 3982393 bp | 10111733 bp |
|  | Promoters | 55 (+ 7 histone genes) | 21 | 58 | 134 |
|  | CpG island-promoters | 28 | 12 | 31 | 71 |
|  | non-CpG island promoters | 27 | 9 | 27 | 63 |
|  | Promoter density | 1/59 kb | 1/137 kb | 1/68 kb | 1/75 kb |
|  | CpG island density | 1/116 kb | 1/240 kb | 1/128 kb | 1/142 kb |
|  | | | | | |
| **300-800 nt**  **NASCENT STRANDS**  **HYBRIDISATION** | ORIs | 34 | 24 | 39 | 97 |
|  | CpG island-ORIs | 15 | 9 | 14 | 38 |
|  | non-CpG island promoter-ORIs | 1 | 2 | 2 | 5 |
|  | Exonic-ORIs | 7 | 1 | 9 | 17 |
|  | 3´UTR-ORIs | 2 | 1 | 2 | 5 |
|  | Intronic-ORIs | 4 | 8 | 5 | 17 |
|  | Intergenic-ORIs | 5 | 3 | 7 | 15 |
|  | ORI density | 1/93 kb | 1/120 kb | 1/102 kb | 1/103 kb |
|  | % of promoter-ORIs | 47% | 46% | 41% | 44% |
|  | | | | | |
| **ORI OCCURRENCE**  **WITH**  **ALGORITHM 1** | % annotated CpG islands with ORI activity | 54% | 75% | 45% | 50% |
|  | % annotated non-CpG islands-promoters with ORI activity | 4% | 22% | 7% | 8% |
|  | % annotated promoters with ORI activity | 29% | 52% | 28% | 32% |
|  | | | | | |
| **ORI OCCURRENCE**  **WITH**  **A LESS**  **STRINGENT CRITERIA**  **(ALGORITHM 2)** | % annotated CpG islands with ORI activity | 82% | 83% | 84% | 83% |
|  | % annotated non-CpG islands-promoters with ORI activity | 22% | 22% | 48% | 33% |
|  | % annotated promoters with ORI activity | 53% | 57% | 67% | 60% |
