# Supplementary material for: Transcription Initiation Activity Sets Replication Origin Efficiency in Mammalian Cells
Source: PLoS Genet. 2009 Apr 10;5(4):e1000446. doi: 10.1371/journal.pgen.1000446 (PMC2661365; doi:10.1371/journal.pgen.1000446)
Supplement: Table S3 — List of the primers used in this work. (0.32 MB DOC) [file pgen.1000446.s004.doc]

**Oligos used for qPCR of array validations:**

| **Region** | **Oligo name** | **Sequence** |
| --- | --- | --- |
| 105455 | 1 | 5’- ATATTTCCCCTCACCGCACC |
|  | 2 | 5’- CTTCAAGAAGCTCCGGGAGC |
|  | 3 | 5’- GATCCTGAACCAGCCGCTGG |
|  | 4 | 5’- GCTGTTGCTCTCCACAGGAG |
|  | 5 | 5’- CGCTTGTTTCCGGCCGGACG |
|  | 6 | 5’- CCAGCGGCTGGTTCAGGATC |
|  | 7 | 5’- GCTGTGTGATCGCACTCTTC |
|  | 8 | 5’- ATCTCAACAGGAGCCACACC |
| 105490 | 1 | 5’- actcttgtatgagtgaatgg |
|  | 2 | 5’- accagtacctactgtaatag |
|  | 3 | 5’- gattccttggagttgacatc |
|  | 4 | 5’- gagtcaagaactttcaaagg |
|  | 5 | 5’- tggggaaatggtgatagatg |
|  | 6 | 5’- ttgcaaagtgttctctggac |
|  | 7 | 5’- ttgatggagctaatgggaag |
|  | 8 | 5’- tccactgagcaaagtttaac |
| 106169 | 1 | 5’- gggtagagcccgtgagttag |
|  | 2 | 5’- tctgggcttagagtaaggag |
|  | 3 | 5’- ccaggggctcagtagaaaag |
|  | 4 | 5’- aggcttgggagaatggttac |
|  | 5 | 5’- tgctctttccattagacagg |
|  | 6 | 5’- ttctgtgggcagtgcccatg |
|  | 7 | 5’- tgagagctggagagtggacg |
|  | 8 | 5’- ccttgccgggaaaacaccgc |
| 106197 | 1 | 5’- GCTATTAAATAAGGCAGTGC |
|  | 2 | 5’- GTATAGTGCTGACTTTAGTGAG |
|  | 3 | 5’- CAATTGGTTCCCGTGCCTCC |
|  | 4 | 5’- CAATCCCCTGGCTCTTGCTC |
|  | 5 | 5’- CCACCGGGATGGAGACGATG |
|  | 6 | 5’- CAGCGCTCGCAAAGTGCCTC |
|  | 7 | 5’- TGGAGACGATGCGAGCACAG |
|  | 8 | 5’- AAAGTGCCTCTCCCACCGAC |
|  | 9 | 5’- ACTGTCCTCACAGTGGCTCC |
|  | 10 | 5’- TCCCCAAAAGGAAATGCATG |
| 106334 | 1 | 5’- tgagtgtcagaacaccgtgg |
|  | 2 | 5’- ccatgaataaactagggagg |
|  | 3 | 5’- agcccttcctggaagcaaag |
|  | 4 | 5’- ggcagttctgacttctgtcc |
|  | 5 | 5’- aagaagcactgccctgtcac |
|  | 6 | 5’- tcttccctgtcccaaagctc |
|  | 7 | 5’- ggtaagtggccatgtttagg |
|  | 8 | 5’- ggaagagcacatcatgtgtc |
| 106402 | 1 | 5’- ctggagtagtactgtaatcc |
|  | 2 | 5’- ttccagcaacaaacagaagc |
|  | 3 | 5’- gaacttagaaccagagtcag |
|  | 4 | 5’- ttatcctacccagccttcag |
|  | 5 | 5’- cttttctttctttgctaggc |
|  | 6 | 5’- acttagagagagggtcactg |
|  | 7 | 5’- tgccagttttcatttgcctc |
|  | 8 | 5’- agacacaccacgcctgtaag |
| 108091 | 1 | 5’- agagtctcgctctgcgattc |
|  | 2 | 5’- tatacgtcagccgtaaaacc |
|  | 3 | 5’- gagctccaggactcagcgag |
|  | 4 | 5’- gacgcagagcgggcatcttc |
|  | 5 | 5’- gcgtgctctaccctgccccc |
|  | 6 | 5’- gtggagacgttgcttgatcc |
|  | 7 | 5’- atatcccaccataacaagcc |
| 108415 | 1 | 5’- cacctgcttcctacacatcc |
|  | 2 | 5’- ttagcagcttgttcctttgg |
|  | 3 | 5’- taactggggttatgatgagg |
|  | 4 | 5’- atcaccgactgtgttctgcc |
|  | 5 | 5’- tcaaaaactgtcactactgc |
|  | 6 | 5’- acagaaaataacgtggtacc |
|  | 7 | 5’- gaaggtgatttggtttgaag |
|  | 8 | 5’- aacactagcaagtttccgaac |
|  | 9 | 5’- agaggccaacatgaatgttc |
|  | 10 | 5’- tgcctctgatctctcaaatg |
|  | 11 | 5’- tcctcctgttagaggaaaag |
|  | 12 | 5’- tgggactctagtagctatcg |
| 108639 | 1 | 5’- ggaagaaagggctatcctcc |
|  | 2 | 5’- atgtcagccacaccaggctg |
|  | 3 | 5’- cgcgaaaagcccttccgctg |
|  | 4 | 5’- gttgcggctgaagcctttgc |
|  | 5 | 5’- ccctacatgtgctccgagtg |
|  | 6 | 5’- ggtggttgacgaggtgcgag |
|  | 7 | 5’- cccaggagatggttaataag |
|  | 8 | 5’- tcaagcacatcttctaagag |
| 109331 | 1 | 5’- gggtactcataggtcagagg |
|  | 2 | 5’- aatttgggaggccacaaggc |
|  | 3 | 5’- agagagtctccaggccctgg |
|  | 4 | 5’- cgccaatcctgggtgaaaag |
|  | 5 | 5’- ttgcatccacgcgcgcaaac |
|  | 6 | 5’- acaaagtgctttgggactgc |
|  | 7 | 5’- tacaggcaggcacactcaag |
|  | 8 | 5’- tgagctgggtgggaatactg |
|  | 9 | 5’- tcggcaggagcttaggttcc |
| 65030 | 1 | 5’- GTGGGGAAAAACAAATCAGG |
|  | 2 | 5’- ATGTCAGGCTTTTCTCTTGCG |
|  | 3 | 5’- ACGCGCCCCTCTCTTCTTAG |
|  | 4 | 5’- TCACCTGCTCTTCCAGCCAG |
|  | 5 | 5’- CCCCTTGGCCCATCTTGCTG |
|  | 6 | 5’- GAAGCGACCTAGGGCTTAGG |
|  | 7 | 5’- CAATTGCGGCCTGGCTATCG |
|  | 8 | 5’- CCACTCTCGAGCGCCAAAAC |
|  | 9 | 5’- CTGCGTGCGCAAACAGGGCG |
|  | 10 | 5’- AAATGGCCTCACCGTTCTCC |
| 65321 | 1 | 5’- tcctcattcagatcacctag |
|  | 2 | 5’- cagattctgggtctcttaac |
|  | 3 | 5’- ggcttggcaaagcacttacc |
|  | 4 | 5’- ttgagggcatcactcctctc |
|  | 5 | 5’- tatgtctcagtctcatctgc |
|  | 6 | 5’- gagtgatgccctcaacagtg |
|  | 7 | 5’- gcaccagtaactggaattgg |
| 65456 | 1 | 5’- ctggctcctgcataagatac |
|  | 2 | 5’- ctacaggtgagatagatgtc |
|  | 3 | 5’- tccctcaaccatacatctcc |
|  | 4 | 5’- tgcctggtgttcaagactag |
|  | 5 | 5’- tcctatctccctgtcaggtc |
|  | 6 | 5’- tctgccccagtttcagtatg |
|  | 7 | 5’- ctcccttgatgacttcagtc |
|  | 8 | 5’- ggttctaggacattggtctc |
| 66347 | 1 | 5’- cttttggatcaatggattgc |
|  | 2 | 5’- caaattctcttgtcttctcc |
|  | 3 | 5’- agcggaggtgatgaattgtg |
|  | 4 | 5’- cagctatgactaacgctacg |
|  | 5 | 5’- aagcgctggccctcacagag |
|  | 6 | 5’- gcgggacagcccaagcgttg |
|  | 7 | 5’- gttggcgcgcgactacacag |
|  | 8 | 5’- cgagttgctgtaccgggtgg |
|  | 9 | 5’- ccaggctctgttgcgccttc |
|  | 10 | 5’- cagtacacgggacacctcac |
| 66836 | 1 | 5’- gcttagcttgatgggatgag |
|  | 2 | 5’- actagaaaggtgaggcgtgc |
|  | 3 | 5’- tgatcctggaacgaatggag |
|  | 4 | 5’- agcaggatggctgctacctg |
|  | 5 | 5’- gacagtgacatcaccgggag |
|  | 6 | 5’- gaaagagagggacagttgga |
|  | 7 | 5’- ctggggccatggcgaaaaag |
|  | 8 | 5’- acacgatgagaggacccttc |
|  | 9 | 5’- agggtagcttccagatgaac |
|  | 10 | 5’- acctcagtggccaatcactc |
| 66946 | 1 | 5’- agcctgcatgtggactgcag |
|  | 2 | 5’- agtctagggagaaggagcag |
|  | 3 | 5’- gcttcctgctccatcctctc |
|  | 4 | 5’- accccatacttactgctagg |
|  | 5 | 5’- cttctccagcctcttcttcc |
|  | 6 | 5’- agacctccagtctcacgctg |
|  | 7 | 5’- cctagtgaaccctctggagc |
|  | 8 | 5’- caggcgttgccaaggtctgc |
|  | 9 | 5’- aaacggtcacctagacaaag |
|  | 10 | 5’- tttctgattctgagttcctc |
| 67065 | 1 | 5’- tgcctatgtcacctggatcg |
|  | 2 | 5’- ataagaacctggcaggcagc |
|  | 3 | 5’- gtcctccctaggtgacattg |
|  | 4 | 5’- tcccatgtctggcaacaaag |
|  | 5 | 5’- gaccttcctgtcattcctcc |
|  | 6 | 5’- ttctgtgcccaccatatacc |
|  | 7 | 5’- cgcacacccatagacatacc |
|  | 8 | 5’- tttcacctggggacctgctc |
|  | 9 | 5’- cctgcaccctacctgttcag |
|  | 10 | 5’- cacctcagccctcttctagg |
|  | 11 | 5’- gcctaggagtgggaccattc |
|  | 12 | 5’- cagggttttcctgctcagtc |
| 67276 | 1 | 5’- TGATGAGAACTACAACCATC |
|  | 2 | 5’- ATGTTCCTAGGTCCCACAAC |
|  | 3 | 5’- TTGTGGCGCACTCTCCCAAC |
|  | 4 | 5’- CACACAGACTGGCGCGCGTG |
|  | 5 | 5’- GAGGGCGTCATTCGAAGGTG |
|  | 6 | 5’- AATCGCGAGCGACGGTTCTC |
|  | 7 | 5’- AGCGGTGACGCCCTCAATTG |
|  | 8 | 5’- CCTCTTTTCCCTGCCTAAAC |
|  | 9 | 5’- CGTTTATAATGGGCAGATGG |
|  | 10 | 5’- TCAACAATGACAGACCGATC |
| 42523 | 1 | 5’- gatgtctgttactttagcac |
|  | 2 | 5’- cttttgaaagtttggcatgg |
|  | 3 | 5’- aagtcaagccaaggccatgg |
|  | 4 | 5’- aggtgggttcaaccggagt |
|  | 5 | 5’- ttacaggtcggggcaatggc |
|  | 6 | 5’- aggctggagctgaaggtttc |
|  | 7 | 5’- gagaccgccagccaataatc |
|  | 8 | 5’- cccagtaacttccagccgag |
|  | 9 | 5’- ctctttattcgagcacactc |
|  | 10 | 5’- gcttggtcattagggctttc |
| 43276 | 1 | 5’- gaagaggttattttggaacc |
|  | 2 | 5’- accttgcttgaataagtcgg |
|  | 3 | 5’- gaaatctagcttctagagtc |
|  | 4 | 5’- actcctcatttcctacaaac |
|  | 5 | 5’- ccagggacttgcttgaacag |
|  | 6 | 5’- tcataacccaggcctgctac |
|  | 7 | 5’- atcatctggcttcatagatc |
|  | 8 | 5’- aagtccaaatttgtggcttg |
| 44450 | 1 | 5’- gaagttcccatgcaattctc |
|  | 2 | 5’- cttcctgccaactttctcag |
|  | 3 | 5’- cagaagcagaaatagggtcc |
|  | 4 | 5’- tactcgcaactcattcattc |
|  | 5 | 5’- atctgaccgctcctagtttc |
|  | 6 | 5’- catttctcagttgtagatgc |
|  | 7 | 5’- tctgcaaaaagcagaaaggc |
|  | 8 | 5’- gtcattatatttgggtgctg |
|  | 9 | 5’- acagaaagcaggaaaacagc |
|  | 10 | 5’- tatcctttcaacaatgtccc |
| 44793 | 1 | 5’- TCCCAGCTTAATTAATCTGG |
|  | 2 | 5’- ACAACAATCACATGTGGTTG |
|  | 3 | 5’- CCAAACTTCAGCAGTTTTCC |
|  | 4 | 5’- TCCTCTTGCAAGACAATTTC |
|  | 5 | 5’- ATTTTTTACTGGCCTCCTTG |
|  | 6 | 5’- AGACAACCACTAGTCTTGTG |
|  | 7 | 5’- TTATCCACTTTCCTCTGTTC |
|  | 8 | 5’- GGGGGAAGGGAAAGTTTAAG |

**Oligos used for ChIP:**

| Region | Oligo name | Sequence |
| --- | --- | --- |
| 105455 | 1 | 5’- ATATTTCCCCTCACCGCACC |
|  | 2 | 5’- CTTCAAGAAGCTCCGGGAGC |
|  | 3 | 5’- GATCCTGAACCAGCCGCTGG |
|  | 4 | 5’- GCTGTTGCTCTCCACAGGAG |
|  | 5 | 5’- CGCTTGTTTCCGGCCGGACG |
|  | 6 | 5’- CCAGCGGCTGGTTCAGGATC |
|  | 9 | 5’- CGACCTTGCCCAGTCACTTC |
|  | 10 | 5’- ACAAGCATCCCCAGCCTCTC |
| 67276 | 11 | 5’- TCCAAATCCTTGGTGGAAAC |
|  | 12 | 5’- GACTGAGATGTTGTAGGACA |
|  | 13 | 5’- TTCAGCTGCGGATTGGTGGA |
|  | 14 | 5’- TGGTGAACTACTCAGCAGGG |
|  | 15 | 5’- TCATACACACCAATCAAGTG |
|  | 16 | 5’- CTGTATGAAAGAGATTGGGT |
| 108639 | 5 | 5’- ccctacatgtgctccgagtg |
|  | 6 | 5’- ggtggttgacgaggtgcgag |
| 106169 | 1 | 5’- gggtagagcccgtgagttag |
|  | R | 5’- caggctgaaggagaggcaag |
| 106334 | 5 | 5’- aagaagcactgccctgtcac |
|  | 6 | 5’- tcttccctgtcccaaagctc |
| 106611 | F | 5’- gctcaagtcagtctcagagg |
|  | R | 5’- cggaccacacacaagaacag |
| 109331 | 7 | 5’- tacaggcaggcacactcaag |
|  | 6 | 5’- acaaagtgctttgggactgc |
| 43276 | 5 | 5’- ccagggacttgcttgaacag |
|  | 6 | 5’- tcataacccaggcctgctac |
| 44450 | F | 5’- ataggctctatttgtagctg |
|  | 8 | 5’- gtcattatatttgggtgctg |
| 65321 | 3 | 5’- ggcttggcaaagcacttacc |
|  | 4 | 5’- ttgagggcatcactcctctc |
| 65456 | F | 5’- actccactggaaaggctcac |
|  | 6 | 5’- tctgccccagtttcagtatg |
| 66946 | 5 | 5’- cttctccagcctcttcttcc |
|  | 6 | 5’- agacctccagtctcacgctg |
| 67065 | 9 | 5’- cctgcaccctacctgttcag |
|  | 10 | 5’- cacctcagccctcttctagg |
| 44793 | 5 | 5’- ATTTTTTACTGGCCTCCTTG |
|  | 6 | 5’- AGACAACCACTAGTCTTGTG |

**Oligos used for luciferase reporter assays:**

| Region | Oligo name | Sequence | Fragment size (bp) |
| --- | --- | --- | --- |
| *Aprt* promoter | Aprt-1 | 5’- GGGGTACCGAAAAAGCGTGTGTGGGGCA | 228 |
|  | Aprt-2 | 5’- GAAGATCTAGGGCGGAGCGTCCTCGATG |  |
| *Notch2*  promoter | 8 | 5’- ttgccctctgccttccgttg | 974 |
|  | 9 | 5’- ggaaagaataacagcagttg |  |
| negative control | 1 | 5’- caacctgccctgcctcaatg | 532 |
|  | 2 | 5’- acacaaatgaggggcctggg |  |
| 108639 | 3 | 5’- cgcgaaaagcccttccgctg | 613 |
|  | 6 | 5’- ggtggttgacgaggtgcgag |  |
| 106334 | 1 | 5’- tgagtgtcagaacaccgtgg | 536 |
|  | 6 | 5’- tcttccctgtcccaaagctc |  |
| 106169 | 9 | 5’- gcactgttgttacaaatagg | 620 |
|  | 4 | 5’- aggcttgggagaatggttac |  |
| 65321 | 1 | 5’- tcctcattcagatcacctag | 816 |
|  | 4 | 5’- ttgagggcatcactcctctc |  |
| 65456 | 3 | 5’- tccctcaaccatacatctcc | 712 |
|  | 9 | 5’- tcactgccttttcagagagg |  |
| 43276 | 5 | 5’- ccagggacttgcttgaacag | 585 |
|  | 8 | 5’- aagtccaaatttgtggcttg |  |
| 44450 | 5 | 5’- atctgaccgctcctagtttc | 709 |
|  | 10 | 5’- tatcctttcaacaatgtccc |  |
| 109331 | 3 | 5’- agagagtctccaggccctgg | 944 |
|  | 10 | 5’- caaagaggtggcctctgagg |  |
| 67065 | 13 | 5’- ttcctccctctgtctgtctc | 493 |
|  | 14 | 5’- agcccaccattccacatagg |  |
